# Activation of the PI3K/mTOR/AKT Pathway and Survival in Solid Tumors: Systematic Review and Meta-Analysis

**DOI:** 10.1371/journal.pone.0095219

**Published:** 2014-04-28

**Authors:** Alberto Ocana, Francisco Vera-Badillo, Mustafa Al-Mubarak, Arnoud J. Templeton, Verónica Corrales-Sanchez, Laura Diez-Gonzalez, María D. Cuenca-Lopez, Bostjan Seruga, Atanasio Pandiella, Eitan Amir

**Affiliations:** 1 Translational Research Unit, Albacete University Hospital, Albacete, Spain; 2 Division of Medical Oncology and Hematology, Princess Margaret Cancer Centre and University of Toronto, Toronto, Canada; 3 Sector of medical Oncology, Institute of Oncology Ljubljana, Ljubljana, Slovenia; 4 Centro de Investigación del Cáncer, CSIC-University of Salamanca, Salamanca, Spain; Lady Davis Institute for Medical Research/McGill University, Canada

## Abstract

**Background:**

Aberrations in the phosphatidylinositol 3-kinase (PI3K)/mammalian target of rapamycin (mTOR)/AKT pathway are common in solid tumors. Numerous drugs have been developed to target different components of this pathway. However the prognostic value of these aberrations is unclear.

**Methods:**

PubMed was searched for studies evaluating the association between activation of the PI3K/mTOR/AKT pathway (defined as PI3K mutation [*PIK3CA*], lack of phosphatase and tensin homolog [PTEN] expression by immunohistochemistry or western-blot or increased expression/activation of downstream components of the pathway by immunohistochemistry) with overall survival (OS) in solid tumors. Published data were extracted and computed into odds ratios (OR) for death at 5 years. Data were pooled using the Mantel-Haenszel random-effect model.

**Results:**

Analysis included 17 studies. Activation of the PI3K/mTOR/AKT pathway was associated with significantly worse 5-year survival (OR:2.12, 95% confidence intervals 1.42–3.16, p<0.001). Loss of PTEN expression and increased expression/activation of downstream components were associated with worse survival. No association between *PIK3CA* mutations and survival was observed. Differences between methods for assessing activation of the PI3K/mTOR/AKT pathway were statistically significant (p = 0.04). There was no difference in the effect of up-regulation of the pathway on survival between different cancer sites (p = 0.13).

**Conclusion:**

Activation of the PI3K/AKT/mTOR pathway, especially if measured by loss of PTEN expression or increased expression/activation of downstream components is associated with poor survival. *PIK3CA* mutational status is not associated with adverse outcome, challenging its value as a biomarker of patient outcome or as a stratification factor for patients treated with agents acting on the PI3K/AKT/mTOR pathway.

## Introduction

Historically, the development of anti-neoplastic drugs has not focused on the targeting of specific molecular aberrations [1]. However, more recently, some targeted drugs have been developed against known oncogenes in selected patient populations. Examples of this are trastuzumab for HER2 over-expressing or amplified breast and gastric cancer [2], [3], imatinib for chronic myeloid leukemia (CML) [4], vemurafenib for metastatic melanoma with (V600E) *B-RAF* mutations and crizotinib for non-small cell lung cancer patients with *anaplastic lymphoma kinase (ALK)* rearrangements [5], [6]. In these examples, development of the drug was carried out in parallel with the identification of a biomarker that permitted the selection of patients with a higher chance of response.

The discovery and validation of biomarkers has become an integral part of successful drug development, with few drugs under development lacking associated biomarker programs. Such biomarkers are typically biological surrogates that can guide in the identification of patients with a higher probability of response. A biomarker can be a known oncogenic alteration like a gene mutation, an overexpressed protein or a protein that reflects the activation status of a signaling pathway [7]. Other types of biomarkers may help monitoring response to treatment or provide information about prognosis and outcome [7]. In this latter case, markers associated with worse outcome can be informative of a more aggressive phenotype potentially guiding the selection of more intensive treatment [8].

**Figure 1:**
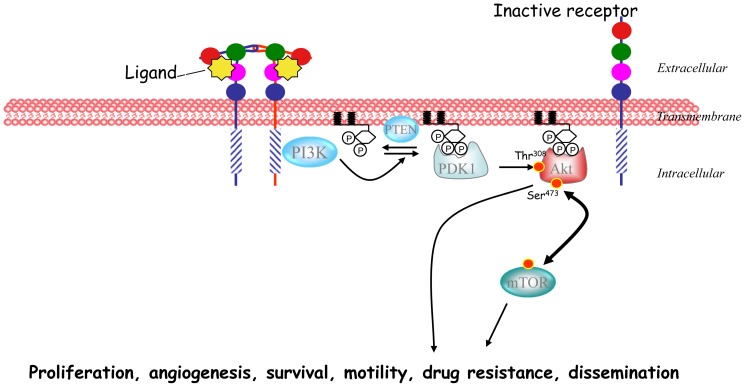
Schematic representation of the PI3K/mTOR pathway.

The phosphatidylinositol 3-kinase (PI3K)/mammalian target of rapamycin (mTOR)/AKT pathway has been linked to the pathophysiology of several neoplastic diseases [9], [10]. Activation of this pathway can be a result of mutations in the *PI3K* or *AKT* genes, loss of phosphatase and tensin homolog (PTEN), or constitutive activation of upstream regulatory pathways such as receptor tyrosine kinases (Figure 1) [9], [10]. Given the pro-oncogenic role of the PI3K/AKT/mTOR pathway in cancer, it has become a target of interest for drug development. Inhibition of mTOR with rapalogs has shown clinical efficacy against some solid tumors, including everolimus for angiomyolipoma associated with tuberous sclerosis, metastatic renal cell carcinoma, breast cancer, or pancreatic neuroendocrine carcinomas and temsirolimus for renal cell carcinoma [11]–[14]. Many other agents in clinical development are designed to inhibit the PI3K/AKT/mTOR pathway at different levels and include pure PI3K inhibitors, dual PI3K-mTOR inhibitors, AKT inhibitors or mTOR inhibitors [15], [16]. Despite the approval of some drugs and the clinical development of other agents targeting the PI3K/AKT/mTOR pathway, little is known about which patients are more likely to benefit from targeting this pathway. Similarly, the relationship between alterations of this pathway and a more aggressive phenotype is unclear.

Here we report a systematic review and meta-analysis of studies assessing the association of activation of the PI3K/AKT/mTOR pathway and clinical outcome in solid tumors.

## Methods

### Identification and selection of studies

This analysis was conducted in line with the Preferred Reporting Items for Systematic Reviews and Meta-Analyses guidelines [17]. Medline (Host: PubMed) was searched for studies published between January 2002 and December 2012, which evaluated the expression of components of the PI3K/AKT/mTOR pathway and survival in solid tumors. We used the MeSH terms “PIK3CA and cancer” and “PIK3CA-mTOR and cancer” and “PTEN loss and cancer” adding the limitation of human studies. In addition we used the entry “PIK3CA or mTOR” and the name of each specific solid tumor (e.g. PIK3CA or mTOR and breast cancer) to recognize additional studies. The search was restricted to publications in English. Additional studies were identified through reviews of citation lists (Figure S1 and figure S2). Eligibility criteria were the availability of survival data for at least 5 years in relation to three types of pathway aberrations; mutations in the *PI3K* gene in any domain as measured by polymerase chain reaction (PCR) or other genomic techniques, the lack of PTEN expression by immunohistochemistry (IHC) or western-blot, or the evaluation of downstream components of the pathway like phospho-S6, mTOR, phospho-mTOR, AKT or phospho-4EBP1 by IHC. Studies reporting outcome of patients who had received a specific targeted agent against the PI3K/AKT/mTOR pathway or related pathways were excluded as were studies reporting only disease free survival or cancer-specific survival.

### Data Extraction

Two authors (VSC and AO) extracted information independently using pre-prepared data abstraction forms. The following details were extracted: tumor type, number of patients, duration of follow-up, mechanism for activation of PI3K/AKT/mTOR pathway (*PI3K* mutation, activation of mTOR/AKT or PTEN loss), methods used for the evaluation of pathway activation, and cut-off used for defining pathway activation. The outcome of interest was five-year overall survival (OS). In all cases, survival data were estimated from Kaplan-Meier curves independently by two authors (FV and MA).

### Data Synthesis

The effect of any aberration in the pathway on overall survival was analyzed initially. Subsequently subgroup analyses were conducted to explore the relationship between survival and different components of the PI3K/AKT/mTOR pathway that were evaluated in each study. Group one was termed “mTOR or AKT activation” and included studies that evaluated downstream components of the pathway including phosphorylated proteins such as mTOR, AKT, S6, and others (see table 1). Group two was termed “*PIK3CA* mutations” and included those studies that evaluated mutations in the *PI3K* gene. The third included studies that evaluated loss of PTEN as measured by IHC. A second subgroup analysis included assessment based on the primary cancer site.

**Table 1:** Characteristics of included studies.

Article	Group Subtype	Tumor Subtype	Follow-up time
**Barbareschi, M ** [21]	PIK3CA mutations	Breast Cancer	Not reported
**Dong, Y ** [26]	PIK3CA mutations	Gynecological cancer	Not reported
**Kalinsky, K ** [27]	PIK3CA mutations	Breast Cancer	Median 12.8 years (range not reported)
**Stemke-Hale, K ** [28]	PIK3CA mutations	Breast Cancer	Not reported
**Li, SY ** [29]	PIK3CA mutations	Breast Cancer	Median 4.2 years (range 0.2–6.5 years)
**Lai, Y ** [30]	PIK3CA mutations	Breast Cancer	Median, 6.4 years (range 0.1–9.3 years)
**Kirkegaard, T ** [31]	mTOR or AKT activation	Breast Cancer	Median 6.5 years (range 0.6–18.4 years)
**Oh, M ** [32]	mTOR or AKT activation	Non-small Cell Lung Cancer	Median 2.9 years (range 0.1–12.7 years)
**Xiao, L ** [33]	mTOR or AKT activation	Gastrointestinal tumors	Median 5.6 years (range 0.02–12.2 years)
**Yu, Z ** [34]	mTOR or AKT activation	Head and Neck Cancer	Mean 3.0 years (range not reported)
**Yu, G ** [35]	mTOR or AKT activation	Gastrointestinal tumors	Mean 3.1 years (1.8–6.1 years)
**Castellvi, J ** [24]	mTOR or AKT activation	Gynecological cancer	Mean 2.6 years (range 2.0–6.7 years)
**Hsu, CP ** [36]	PTEN loss	Gastrointestinal tumors	Median 4.3 years (range 0.3–6.6 years)
**Lotan, LT ** [37]	PTEN loss	Prostate Cancer	Median 16.0 years (range not reported)
**Sawai, H ** [38]	PTEN loss	Gastrointestinal tumors	Median 3.0 years (range not reported)
**Sze, KM ** [39]	PTEN loss	Gastrointestinal tumors	Not reported
**Terakawa, N ** [40]	PTEN loss	Gynecological cancer	Not reported

### Statistical analysis

The proportion of patients surviving 5 years was estimated from the Kaplan-Meier curves for both normal (control group) and the presence of the molecular alteration (experimental group). The relative frequency of survival at 5 years between the control and experimental groups was expressed as an odds ratio (OR) and its 95% confidence interval (CI). Data were combined into a meta-analysis using RevMan 5.1 analysis software (Cochrane Collaboration, Copenhagen, Denmark). Estimates of ORs were weighted and pooled using the Mantel-Haenszel method. Cochran's Q (p<0.10) and the I^2^ index (>50%) were used to define inter-study heterogeneity. Due to significant heterogeneity, random effects modeling was used for all analyses. Analyses were conducted for all studies and differences between the subgroups were assessed using methods described by Deeks et al. [18]. All statistical tests were two sided, and statistical significance was defined as p<0.05. No corrections were made for multiple comparisons.

## Results

### Description of studies

We identified 17 studies that evaluated activation of the PI3K/mTOR/AKT pathway and survival in solid tumors. These studies comprised a total of 4746 patients with a median sample size of 279 patients. The characteristics of included studies are shown in table 1. Six studies evaluated the expression of the PI3K/AKT/mTOR pathway in breast cancer, five in gastrointestinal tumors, three in gynecological cancers, and one each in prostate, non-small cell lung cancer (NSCLC) and oropharyngeal cancers. Six studies were included in the group called “mTOR or AKT activation”, six in the “*PIK3CA* mutation” group and five in the “PTEN loss” group. The estimated median follow-up was 4.3 years (range = 2.6 to 16 years). The prevalence of pathway activation and the methods used for the analyses of molecular alterations of this pathway are shown in Table 2.

**Table 2:** Prevalence and analyses of the molecular alterations of the PI3K/mTOR/AKT pathway.

Article	Prevalence	Gene/protein	Method used	Cut-off or staining used
**Barbareschi, M (21)**	N: 163; 46 missense mutation. 24 (53%) in exon 9 (Helicoidal); 21 (47%) in exon 20 (Kinase)	Exon 9: 12 E542K 11 E545K Exon 20: 20 H1047R	PCR amplification of exons 9 and 20 with flanking intronic sequences and single-strand conformation polymorphism (SSCP) followed by sequencing of positive cases	Both are included in the Kaplan-maier. Exon 9, helical is poor prognosis
**Dong, Y (26)**	N: 94; 29 (30.9%)	Exon 9: 28; Exon 20: 16	PCR amplifications of exon 9 and 20. Procuts were sequenced and detected by capillary electrophoresis.	Exon 9 E542G, A1625G; Exon 20 stp1060R, T3205A. No association with survival
**Kalinsky, K (27)**	N: 590; 32.5%	PIK3CA mutations all cases for the three HS mutations	Mutation detection by Sequenom MassARRAY system. The iPLEX Gold Genotyping assay was used	Patients with PIK3CA H1047R mutated tumors have significant improvement in overall survival (P = 0.03) and breast cancer-specific survival (P = 0.004)
**Stemke-Hale, K (28)**	PIK3CA mutation 34.5%; HER2: 22.7%; Basal-like tumors: 8.3%	23 known mutations in P3KCA	PCR and Mass spectroscopy based approach evaluating single nucleotide polymorfisms	No difference in kinase domain versus all other (mainly helical domain)
**Li, SY (29)**	N: 250 (35%); Exon 7: 3%; Exon 9: 16%; Exon 20: 19%	PIK3CA mutations reported in human cancer occur in exon 7, 9 and 20	Using PCR and fluorescen t(F)-SSCP	PIK3CA normal versus mutation (exon 7, 9, 20)
**Lai, Y (30)**	N: 152 (26%) More than half in exon 20	Mutations of PIK3CA reported in exon 4 (codon N345I, N345K), 7 (Codon C420R), 9 (E542K, E545A, E545G, E545G, E545K, Q546E), 20 (Codon H1047L, H1047R, H1047Y, H1047L, G1049R)	CR (in thermal cycler GeneAmp PCR System 9700). PCR products were sequenced using the ABI PRISM BigDye Terminator v3.1 cycle sequencing kit and 3730 DNA Analyzer	PIK3CA exon 20 mutations were independent risk factors for overall survival.
**Kirkegaard, T (31)**	N: 402 ER pos. Breast cancers.	AKT (pAKTSer-473)	pan-AKT(AbcamLid, Cambridge,UK); pAKT (Thr-308)(Cell Signallic Tecnology, beverly, USA); pAKT(Ser-473)(Biosource International Inc, CA,USA); Antibody specificity was checked by western blotting using a standard protocol	The cut-off value for high and low levels of pAKT expression is defined as above and below the median histoscore.
**Oh, M (32)**	N: 574 mTOR: Score 0: 22%, Score 2: 13%, Score 3: 11%, Score 4: 15%, Scored 5: 17%, Score 6: 22%.	mTor and pAKT	mTOR staining: incubated with a rabbit monoclonal antibody against mTOR (1∶100, clone 49F9). AKT: rabbit monoclonal antibody against pAkt (1∶50, clone 736E11) Evaluated: Tumor cells were judged as positive for pAkt if membranous, cytoplasmic and/or nuclear staining was present.	A semiquantitative immunohistochemical score for intensity of staining and the extent of staining. Intensity, a score of 0 to 3 (corresponding to negative, weak, moderate, and strong positivity) Extent of staining was scored as 0 (0%), 1 (1–10%), 2 (11–50%), and 3 (51–100%), The sum of the intensity and extent score was used as the final score (0–6). Final score ≥3: positive.
**Xiao, L (33)**	412 gastric carcinomas, 47 adenomas, 197 non-neoplastic mucosa. mTOR: 66.3% of non-neoplastic mucosa, 70.1% adenomas,61.2% gastric carcinomas.	m-TOR , pS6	Rabbit anti-mTOR antibody (Clone ID: Y392, 1612-1, Epitomics, USA; 1∶250) Evaluated: anti-phospho-p70 s6 kinase (pT389, Clone ID; E175, 1175-1, Epitomics, USA; 1∶50) followed by exposure to the anti-rabbit Envison-PO(DAKO, USA) antibody	The positive percentage of counted cells was graded semi-quantitatively according to a four-tier scoring system; negative 0–5%; weakly positive 6–25%, moderate positive 26–50%, strongly positive 51–100%.
**Yu, Z (34)**	p-AKT 18%	p-Akt	Primary antibody to p-Akt (ser 473) Cell Signaling Technology (Beverly, MA)	AQUA scores for nuclear and cytoplasmic p-Akt. The lowest quartile was compared with the rest of the cohort.
**Yu, G (35)**	m-TOR: 50.8%; p-mTOR: 46,5%	mTOR, p-mTOR. (prognostic factor pmTOR)	mTOR (dilution 1∶50; clone Y391; Abcam), Evaluated: p-mTOR (Ser 2448; dilution, 1∶100; clone 49F9;CST)	A semiquantitative scoring system was used. An underexpression was defined as no staining or staining positivity in tumor tissue being less than matched normal tissue, a normal expression as staining positivity being similar to matched normal tissue, overexpression as staining positivity being higher than normal tissue.
**Castellvi, J (24)**	p-4EBP1 (47.1%)	p-4EBP1	4EBP1 Cell signaling Tech	Scored the percentage of positive cells and intensity of the staining, which was assessed semiquantitatively. Samples that showed any positivity were grouped together for statistical purposes
**Hsu, CP (36)**	N: 133 CRC group: 89.2% → 53.4%	PTEN	Primary anti-PTEN anti- body (1/200) at room temperature for 2 h	Positive: more than 10%
**Lotan, LT (37)**	N: 397 146 PTEN loss (36.8%).	PTEN	Rabbit monoclonal anti-PTEN antibody(clone D4.3,-9188, cell Signaling Technologies	Using this system, each spot of tumor tissue was scored as negative positive for PTEN protein by comparing staining in malignant gland with that of adjacent benign gland and/or stroma which provided an internal positive control within each tissue core. Staining was classified as negative if the intensity was markedly decreased or entirely negative
**Sawai, H (38)**	PTEN strongly expressed in 62,9% colorectal cancer	PTEN	Anti-PTEN antibodies (clone 28116; Santa Cruz Biotechnology, Santa Cruz, CA,USA	The intensity of tissue staining was graded semi quantitatively on a 4 point scale (−,+,++,+++). Likewise, the proportion of cells stained was assessed on a 4 point scale (1: 0–15%; 2: 25–50%; 3: 50–85% and 4: 85–100% cell stained).Tissues were classified into strongly staining and weakly staining
**Sze, KM (39)**	47,5% PTEN underexpression	PTEN	Cell signalling biotechnology, Denver; MA	Western-blot
**Terakawa, N (40)**	103 endometrial cancers, 36% negative PTEN	PTEN	A mouse monoclonal anti PTEN antibody, PTEN A2B1(Santa Cruz Biotechnology, Santa Cruz CA,USA)	A positive case was defined as one in which all of the tumor cells were stained, a heterogeneous case was defined as one with both staining and non-staining tumor cells and a negative case was defined as one with no staining of any tumor cells

### Association of activation of PI3K/AKT/mTOR pathway and survival

**Figure 2:**
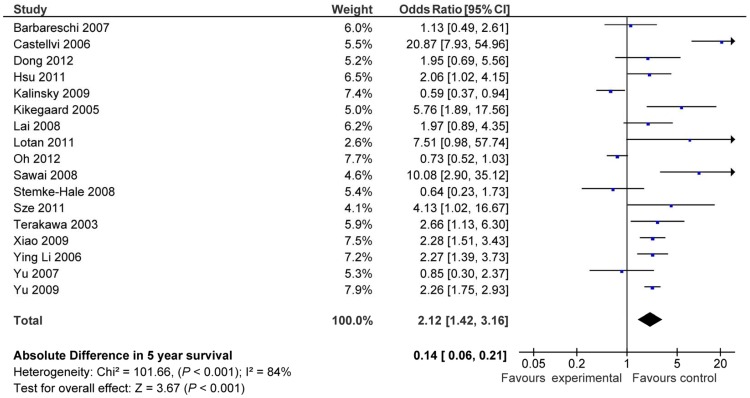
Odds ratio (OR) for 5-year overall survival (OS) in all studies. Forest plots of odds ratios for overall survival at 5 years based on activation of the PI3K/mTOR/AKT pathway. Odds ratios for each trial are represented by the squares, the size of the square represents the weight of the trial in the meta-analysis, and the horizontal line crossing the square represents the 95% confidence interval. The diamonds represent the estimated pooled effect based for each cohort individually (labeled subtotal) and for all cohorts together (labeled total).

Overall, there was an association between the presence of molecular alterations in the PI3K/AKT/mTOR pathway and worse 5-year survival (OR 2.12; 95% CI 1.42–3.16, p<0.001) (Figure 2). There was significant inter-study heterogeneity (Cochran's Q p<0.001, I^2^ = 84%).

### Association of pathway activation and survival by tumor type

Studies in gastrointestinal tumors (n = 5) and gynecologic cancers (n = 3) showed a numerically higher association with worse survival (OR 2.51; 95% CI 1.83–3.43 and OR 4.78; 95% CI 1.14–20.1, respectively) compared with studies in breast cancer (n = 6) and other solid tumors (NSCLC, prostate and oropharyngeal, [n = 3]) which showed no association (OR 1.43; 95% CI 0.74–2.79 and OR 1.08; 95% CI 0.44–2.67, respectively). Of interest, in the one study in prostate cancer, there was a large magnitude of effect on survival (OR 7.51, 95% CI 0.98–57.74), but there was no obvious effect seen in studies of NSCLC and orophayngeal cancer (OR 0.73, 95% 0.52–1.03 and 0.85, 95% CI 0.30–2.37, respectively). However, these differences did not meet statistical significance (subgroup difference p = 0.13).

### Association of pathway activation and survival by type of activation

**Figure 3:**
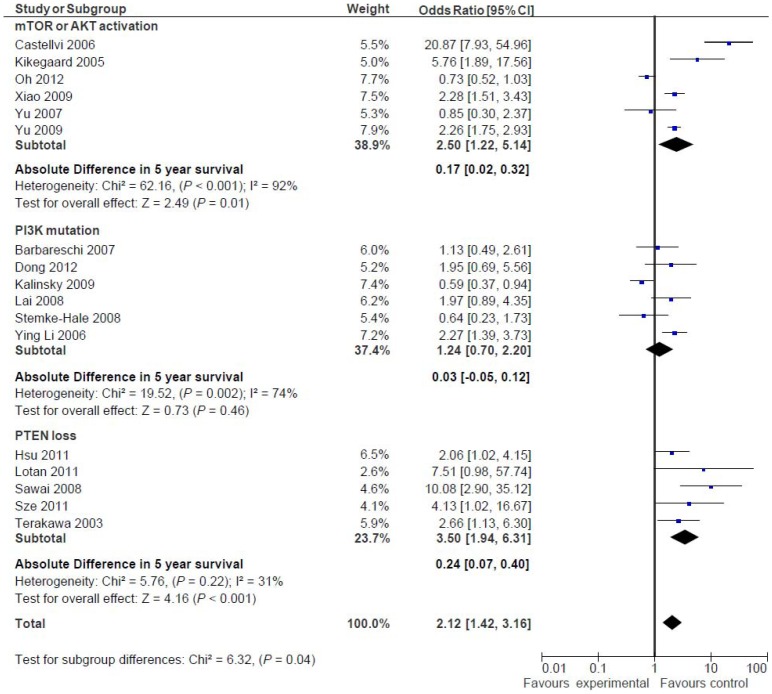
Odds ratio (OR) for 5-year overall survival (OS) according to the expression of different components of the PI3K/mTOR pathway (group subtype). Forest plots of odds ratios for overall survival at 5 years split by subgroups defined by type of activation of the PI3K/mTOR/AKT pathway. Odds ratios for each trial are represented by the squares, the size of the square represents the weight of the trial in the meta-analysis, and the horizontal line crossing the square represents the 95% confidence interval. The diamonds represent the estimated pooled effect based for each cohort individually (labeled subtotal) and for all cohorts together (labeled total).

The results of the subgroup analysis based on the specific part of the PI3K/AKT/mTOR pathway are shown in figure 3. For studies assessing *PIK3CA* gene mutations (n = 6), there was no association with worse 5-years survival (OR: 1.24; 95% CI 0.70–2.20, p = 0.46). In contrast, among studies evaluating activated components of mTOR or AKT (n = 6) a significant association with worse outcome was observed (OR: 2.50; 95% CI 1.22–5.14, p = 0.01). Similarly, studies assessing PTEN loss by IHC (n = 5) showed a significant association with worse survival (OR 3.50; 95% CI 1.94–6.31, p<0.001). This difference between subgroups was significant (p = 0.04).

## Discussion

The identification of biomarkers that can inform the clinical behavior of a given tumor is important for patient education and treatment planning. In this study we explored the prognostic role of different components of the PI3K/AKT/mTOR pathway with the intention to identify tumors that rely on this molecular alteration. Such information may help to guide the clinical development of therapeutic strategies against this pathway.

Overall, there was evidence of an association between alterations in the PI3K/AKT/mTOR pathway and poor survival. When analyzing different components of the pathway we observed that those studies evaluating loss of PTEN and activated components of downstream proteins were linked with the poorest 5-years survival. Conversely, *PIK3CA* mutations were not linked with worse outcome in our analysis. The fact that PTEN is a major regulator of the activation of the PI3K pathway could explain its association with worse outcome [19], [20] as those tumors with loss of PTEN expression could have activation of the different components of this pathway. In contrast, although *PIK3CA* mutations are considered driver mutations because they are linked to cell survival and increased proliferation, in our study there was no association with poor outcome [9], [10]. *PIK3CA* mutations compromise numerous molecular lesions including those affecting both the catalytic and helical domains. Mutations in the helical domain may favor the oncogenic capability of *PIK3CA* by facilitating its interaction with certain signaling intermediates linked to the transmission of pro-oncogenic signals [21]. In the individual studies included in our analysis, mutations at different domains were pooled and this could decrease the statistical power needed to detect a worse outcome in this group for specific mutations. In addition, mutations can have different functional roles and different clinical behavior depending on the tumor type. For example, recent studies have shown that *PIK3CA* mutations are associated with different outcomes in breast cancer depending on whether the tumor is estrogen receptor positive or negative, and whether HER2 is over-expressed or amplified compared to HER2-normal [22], [23]. Regardless of this, the data presented challenges the clinical relevance of *PIK3CA* mutations as unique measures of PI3K/AKT/mTOR pathway activation. This is relevant, as some ongoing clinical trials with agents that target this pathway are using such mutational analysis as an indication of pathway activity, with mutations being used as biomarkers for selection of patients undergoing experimental treatments with PI3K inhibitors.

The analysis of studies evaluating downstream components by IHC showed a significant association with worse survival. These findings have substantial clinicopathological relevance, as evaluation of the activity of a protein appears more biologically relevant than the estimation of gene expression. Therefore, assessment of phosphorylated forms of signaling surrogates such as pS6 or pAKT may be more precise than evaluation of their total levels. However, results from these studies were heterogeneous as the markers evaluated belong to different components of the pathway (mTORC1 and mTORC2) [24].

These data may have relevance beyond prognostic value. Among solid tumors where targeted therapy has been developed against a known oncogene, presence of the oncogene has generally been associated with worse outcome. This is the case in HER2/neu over-expressing or amplified breast or gastric cancers or BRAF-mutated melanoma. Consequently, it is possible that the effect of drugs targeting the PI3K/AKT/mTOR pathway will only be seen in patients where biomarkers consistently show a detrimental clinical outcome. Based on this hypothesis, it would be expected that PIK3CA mutations may not be associated with improvement in outcome from drugs targeting the PI3K/AKT/mTOR pathway. This hypothesis is supported by data in breast cancer, which show little predictive value of PIK3CA with the mTOR inhibitor everolimus [15], [16], [25]. On the other hand, it is known that not all druggable molecular alterations in cancer are linked with worse outcome like the expression of estrogen receptors in breast cancer.

When analyzing the results by tumor type, alterations of the PI3K/AKT/mTOR pathway in breast cancer were not linked with worse outcome. However, most of these studies evaluated *PIK3CA* mutations. Conversely, studies in gynecological tumors and gastrointestinal cancers were more enriched in studies evaluating PTEN and protein markers of “mTOR or AKT activation”; and these were linked with worse outcome. Only single studies in NSCLC, oropharyngeal and prostate cancers were available and these showed variable results. These studies were generally small and consequently reported wide confidence intervals which crossed the null boundary. Consequently, the relevance of these results in isolation remains unclear. The inconsistent measurement of pathway activation means that the independent effect of PI3K/mTOR/AKT activation in different tumor types cannot be evaluated with certainty

Our study has limitations. This is a meta-analysis of the literature and is therefore more likely to be compromised by selection bias with enrichment for studies reporting positive results. In addition, there is also substantial intra- and inter-study heterogeneity including differences in biomarkers of interest and variability in *PIK3CA* domain mutations. Despite the use of statistical methods to reduce the effects of such heterogeneity, there remains uncertainty regarding the accuracy of the pooled estimates. Furthermore, hazard ratios were not reported by most studies and therefore we estimated the odds of death at 5 years instead. This is a less robust measure for survival, but was the only feasible method using the available data.

Finally, despite these limitations, results of this study do have some implications for both clinical and translational research. It is shown that the activation of the PI3K/AKT/mTOR pathway is related to poor outcome, and it is particularly relevant in gastrointestinal and gynecological cancers. In addition, the evaluation of PTEN levels ideally complemented with concomitant evaluation of the activation status of proteins such as pS6 and AKT is linked with worse outcome probably identifying tumors that rely most on the PI3K/AKT/mTOR pathway.

## Supporting Information

Figure S1 **Flow diagram of literature search.**
(TIFF)

Figure S2 **PRISMA flowchart using MeSH terms.**
(PPT)

Checklist S1 (DOC)

## References

[1] Amir E, Seruga B, Martinez-Lopez J, Kwong R, Pandiella A, et al (2011) Oncogenic targets, magnitude of benefit, and market pricing of antineoplastic drugs. J Clin Oncol 29: 2543–2549. 21606435 10.1200/JCO.2011.35.2393

[2] Slamon DJ, Leyland-Jones B, Shak S, Fuchs H, Paton V, et al (2001) Use of chemotherapy plus a monoclonal antibody against HER2 for metastatic breast cancer that overexpresses HER2. N Engl J Med 344: 783–92. 11248153 10.1056/NEJM200103153441101

[3] Bang YJ, Van Cutsem E, Feyereislova A, Chung HC, Shen L, et al (2010) Trastuzumab in combination with chemotherapy versus chemotherapy alone for treatment of HER2-positive advanced gastric or gastro-oesophageal junction cancer (ToGA): a phase 3, open-label, randomised controlled trial. Lancet 376: 687–97. 20728210 10.1016/S0140-6736(10)61121-X

[4] O'Brien SG, Guilhot F, Larson RA, Gathmann I, Baccarani M, et al (2003) Imatinib compared with interferon and low-dose cytarabine for newly diagnosed chronic-phase chronic myeloid leukemia. N Engl J Med 348: 994–1004. 12637609 10.1056/NEJMoa022457

[5] Chapman PB, Hauschild A, Robert C, Haanen JB, Ascierto P, et al (2011) Improved survival with vemurafenib in melanoma with BRAF V600E mutation. N Engl J Med 364: 2507–16. 21639808 10.1056/NEJMoa1103782 PMC3549296

[6] Kwak EL, Bang YJ, Camidge DR, Shaw AT, Solomon B, et al (2010) Anaplastic lymphoma kinase inhibition in non-small-cell lung cancer. N Engl J Med 363: 1693–703. 20979469 10.1056/NEJMoa1006448 PMC3014291

[7] Sawyers CL (2008) The cancer biomarker problem. Nature 452: 548–52. 18385728 10.1038/nature06913

[8] Paik S, Shak S, Tang G, Kim C, Baker J, et al (2004) A multigene assay to predict recurrence of tamoxifen-treated, node-negative breast cancer. N Engl J Med 351: 2817–26. 15591335 10.1056/NEJMoa041588

[9] Engelman JA (2009) Targeting PI3K signalling in cancer: opportunities, challenges and limitations. Nat Rev Cancer 9: 550–62. 19629070 10.1038/nrc2664

[10] Courtney KD, Corcoran RB, Engelman JA (2010) The PI3K pathway as drug target in human cancer. J Clin Oncol 28: 1075–83. 20085938 10.1200/JCO.2009.25.3641 PMC2834432

[11] Bissler JJ, Kingswood JC, Radzikowska E, Zonnenberg BA, Frost M, et al (2013) Everolimus for angiomyolipoma associated with tuberous sclerosis complex or sporadic lymphangioleiomyomatosis (EXIST-2): a multicentre, randomised, double-blind, placebo-controlled trial. Lancet 381: 817–24. 23312829 10.1016/S0140-6736(12)61767-X

[12] Motzer RJ, Escudier B, Oudard S, Hutson TE, Porta C, et al (2008) Efficacy of everolimus in advanced renal cell carcinoma: a double-blind, randomised, placebo-controlled phase III trial. Lancet 372: 449–56. 18653228 10.1016/S0140-6736(08)61039-9

[13] Baselga J, Campone M, Piccart M, Burris HA 3rd, Rugo HS, et al (2012) Everolimus in Postmenopausal Hormone-Receptor-Positive Advanced Breast Cancer. N Engl J Med 366: 520–9. 22149876 10.1056/NEJMoa1109653 PMC5705195

[14] Hudes G, Carducci M, Tomczak P, Dutcher J, Figlin R, et al (2007) Temsirolimus, interferon alfa, or both for advanced renal-cell carcinoma. N Engl J Med 356 2271–81. 17538086 10.1056/NEJMoa066838

[15] Ocana A, Amir E, Seruga B, Martin M, Pandiella A (2013) The evolving landscape of protein kinases in breast cancer: Clinical implications. Cancer Treat Rev 39: 68–76. 22703833 10.1016/j.ctrv.2012.05.004

[16] Garcia-Echeverria C, Sellers WR (2008) Drug discovery approaches targeting the PI3K/Akt pathway in cancer. Oncogene 27: 5511–26. 18794885 10.1038/onc.2008.246

[17] Liberati A, Altman D, Tetzlaff J (2009) The PRISMA statement for reporting systematic reviews and meta-analyses of studies that evaluate health care interventions: explanation and elaboration. PLoS Med 21; 67: e1000100. 10.1371/journal.pmed.1000100 PMC2707010 19621070

[18] Deeks JJ, Higgins JPT, Altman D.G, editors Ltd (2006) Analysing and presenting results. Cochrane Handbook for Systematic Reviews of Interventions 4 2 5 In: The Cochrane Library, Chichester, UK, John Wiley & Sons.

[19] Li J, Yen C, Liaw D, Podsypanina K, Bose S, et al (1997) PTEN, a putative protein tyrosine phosphatase gene mutated in human brain, breast, and prostate cancer. Science 275: 1943–7. 9072974 10.1126/science.275.5308.1943

[20] Alonso A, Sasin J, Bottini N, Friedberg I, Friedberg I, et al (2004) Protein tyrosine phosphatases in the human genome. Cell 117: 699–711. 15186772 10.1016/j.cell.2004.05.018

[21] Barbareschi M, Buttitta F, Felicioni L, Cotrupi S, Barassi F, et al (2007) Different prognostic roles of mutations in the helical and kinase domains of the PIK3CA gene in breast carcinomas. Clin Cancer Res 13: 6064–9. 17947469 10.1158/1078-0432.CCR-07-0266

[22] Baselga J, Cortés J, Im S-A, Clark E, Kiermaier A, et al (2012) Biomarker Analyses in CLEOPATRA: A Phase III, Placebo-Controlled Study of Pertuzumab in HER2-Positive, First-Line Metastatic Breast Cancer (MBC). Cancer Research December 15, 2012; Volume 72, Issue 24, Supplement 3.

[23] Ramirez-Ardila D, Helmijr JC, Look MP, Lurkin I, Ruigrok-Ritstier K, et al (2013) Hotspot mutations in PIK3CA predict treatment outcome on Aromatase Inhibitors but are not predictive for Tamoxifen. Breast Cancer Res Treat 139: 39–49. 23592373 10.1007/s10549-013-2529-7

[24] Castellvi J, Garcia A, Rojo F, Ruiz-Marcellan C, Gil A, Baselga J, et al (2006) Phosphorylated 4E binding protein 1: a hallmark of cell signaling that correlates with survival in ovarian cancer. Cancer 107: 1801–11. 16983702 10.1002/cncr.22195

[25] Hortobagyi GN, Piccart-Gebhart MJ, Hope Rugo S, Howard Burris A, Campone Mario, et al (2013) Correlation of molecular alterations with efficacy of everolimus in hormone receptor–positive, HER2-negative advanced breast cancer: Results from BOLERO-2. J Clin Oncol 31 (suppl; abstr LBA509)

[26] Dong Y, Yang X, Wong O, Zhang X, Liang Y, et al (2011) PIK3CA mutations in endometrial carcinomas in Chinese women: phosphatidylinositol 3′-kinase pathway alterations might be associated with favorable prognosis. Hum Pathol 43: 1197–205. 22209294 10.1016/j.humpath.2011.08.021

[27] Kalinsky K, Jacks LM, Heguy A, Patil S, Drobnjak M, et al (2009) PIK3CA mutation associates with improved outcome in breast cancer. Clin Cancer Res 15: 5049–59. 19671852 10.1158/1078-0432.CCR-09-0632

[28] Stemke-Hale K, Gonzalez-Angulo AM, Lluch A, Neve RM, Kuo WL, et al (2008) An integrative genomic and proteomic analysis of PIK3CA, PTEN, and AKT mutations in breast cancer. Cancer Res 68: 6084–91. 18676830 10.1158/0008-5472.CAN-07-6854 PMC2680495

[29] Li SY, Rong M, Grieu F, Iacopetta B (2006) PIK3CA mutations in breast cancer are associated with poor outcome. Breast Cancer Res Treat 96: 91–5. 16317585 10.1007/s10549-005-9048-0

[30] Lai YL, Mau BL, Cheng WH, Chen HM, Chiu HH, et al (2008) PIK3CA exon 20 mutation is independently associated with a poor prognosis in breast cancer patients. Ann Surg Oncol 15: 1064–9. 18183466 10.1245/s10434-007-9751-7

[31] Kirkegaard T, Witton CJ, McGlynn LM, Tovey SM, Dunne B, et al (2005) AKT activation predicts outcome in breast cancer patients treated with tamoxifen. J Pathol 207: 139–46. 16088978 10.1002/path.1829

[32] Oh MH, Lee HJ, Yoo SB, Xu X, Choi JS, et al (2012) Clinicopathological correlations of mTOR and pAkt expression in non-small cell lung cancer. Virchows Arch 460: 601–9. 22562131 10.1007/s00428-012-1239-6

[33] Xiao L, Wang YC, Li WS, Du Y (2009) The role of mTOR and phospho-p70S6K in pathogenesis and progression of gastric carcinomas: an immunohistochemical study on tissue microarray. J Exp Clin Cancer Res 28: 152. 20003385 10.1186/1756-9966-28-152 PMC2797794

[34] Yu Z, Weinberger PM, Sasaki C, Egleston BL, Speier WF 4th, et al (2007) Phosphorylation of Akt (Ser473) predicts poor clinical outcome in oropharyngeal squamous cell cancer. Cancer Epidemiol Biomarkers Prev 16: 553–8. 17372251 10.1158/1055-9965.EPI-06-0121

[35] Yu G, Wang J, Chen Y, Wang X, Pan J, et al (2009) Overexpression of phosphorylated mammalian target of rapamycin predicts lymph node metastasis and prognosis of chinese patients with gastric cancer. Clin Cancer Res 15: 1821–9. 19223493 10.1158/1078-0432.CCR-08-2138

[36] Hsu CP, Kao TY, Chang WL, Nieh S, Wang HL, Chung YC (2011) Clinical significance of tumor suppressor PTEN in colorectal carcinoma. Eur J Surg Oncol 37: 140–7. 21194879 10.1016/j.ejso.2010.12.003

[37] Lotan TL, Gurel B, Sutcliffe S, Esopi D, Liu W, et al (2011) PTEN protein loss by immunostaining: analytic validation and prognostic indicator for a high risk surgical cohort of prostate cancer patients. Clin Cancer Res 17: 6563–73. 21878536 10.1158/1078-0432.CCR-11-1244 PMC3195839

[38] Sawai H, Yasuda A, Ochi N, Ma J, Matsuo Y, et al (2008) Loss of PTEN expression is associated with colorectal cancer liver metastasis and poor patient survival. BMC Gastroenterol 26; 8: 56. 10.1186/1471-230X-8-56 PMC2611992 19036165

[39] Sze KM, Wong KL, Chu GK, Lee JM, Yau TO, et al (2011) Loss of phosphatase and tensin homolog enhances cell invasion and migration through AKT/Sp-1 transcription factor/matrix metalloproteinase 2 activation in hepatocellular carcinoma and has clinicopathologic significance. Hepatology 53: 1558–69. 21520171 10.1002/hep.24232

[40] Terakawa N, Kanamori Y, Yoshida S (2003) Loss of PTEN expression followed by Akt phosphorylation is a poor prognostic factor for patients with endometrial cancer. Endocr Relat Cancer 10: 203–8. 12790783 10.1677/erc.0.0100203

