# Supplementary figures and images for: Activation of the PI3K/mTOR/AKT Pathway and Survival in Solid Tumors: Systematic Review and Meta-Analysis
Source: PLoS One. 2014 Apr 28;9(4):e95219. doi: 10.1371/journal.pone.0095219 (PMC4002433; doi:10.1371/journal.pone.0095219)

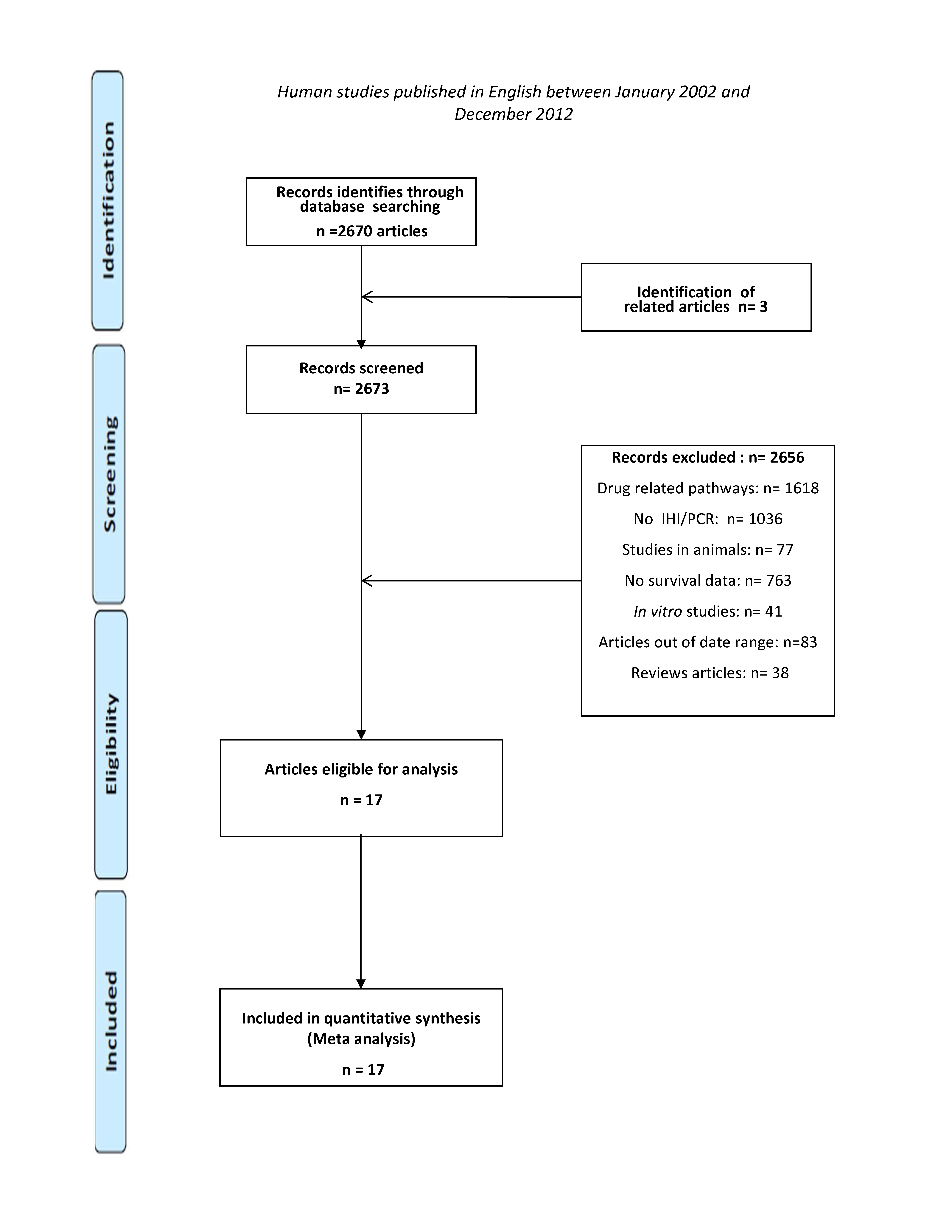

Supplement: Figure S1 — Flow diagram of literature search. (TIFF) [file pone.0095219.s001.tiff]
