# Supplementary material for: Activation of the PI3K/mTOR/AKT Pathway and Survival in Solid Tumors: Systematic Review and Meta-Analysis
Source: PLoS One. 2014 Apr 28;9(4):e95219. doi: 10.1371/journal.pone.0095219 (PMC4002433; doi:10.1371/journal.pone.0095219)
Supplement: Figure S2 — PRISMA flowchart using MeSH terms. (PPT) [file pone.0095219.s002.ppt]

## Slide 1
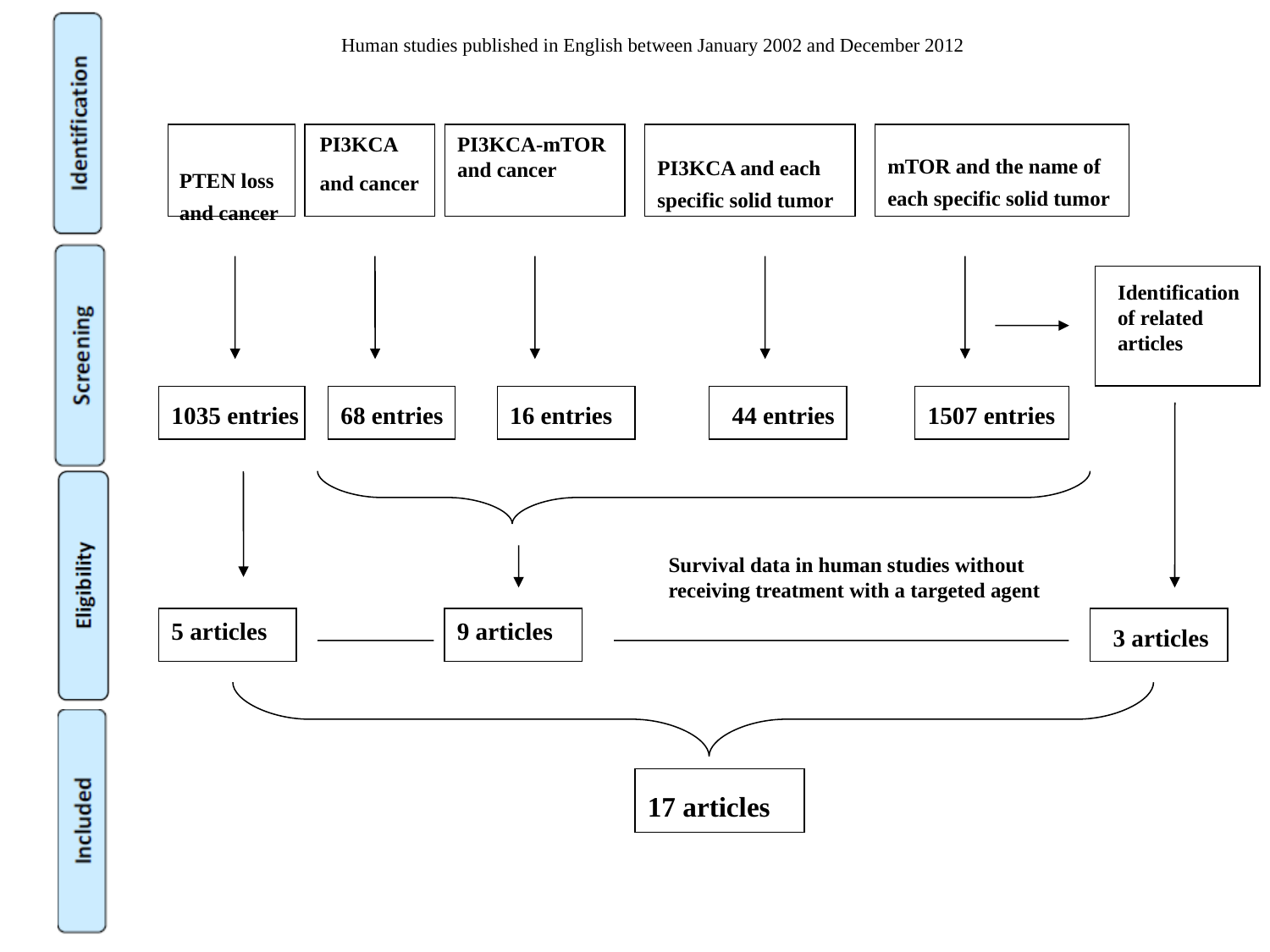

Human studies published in English between January 2002 and December 2012
mTOR and the name of each specific solid tumor
PI3KCA and each specific solid tumor
PTEN loss and cancer
PI3KCA
and cancer
PI3KCA-mTOR and cancer
Identification of related articles
1035 entries
68 entries
16 entries
44 entries
1507 entries
Survival data in human studies without receiving treatment with a targeted agent
5 articles
9 articles
3 articles
17 articles
